# Development of Visible Spectrophotometric Methods for the Determination of Tricyclic Antidepressants Based on Formation of Molecular Complexes with *p*-Benzoquinones

**DOI:** 10.3390/ijms242316744

**Published:** 2023-11-25

**Authors:** Maria D. Ciuca, Radu C. Racovita

**Affiliations:** Department of Inorganic Chemistry, Physical Chemistry and Electrochemistry, Faculty of Chemical Engineering and Biotechnologies, National University of Science and Technology POLITEHNICA Bucharest, 1-7 Gh. Polizu St., District 1, 011061 Bucharest, Romania; maria_daniela.ciuca@upb.ro

**Keywords:** tricyclic antidepressants, *p*-benzoquinones, UV-VIS spectrophotometry, molecular charge transfer complexes, method development, quality control

## Abstract

Tricyclic antidepressants are commonly employed in the management of major depressive disorders. The present work describes two visible (VIS) spectrophotometric techniques that utilize the formation of charge transfer complexes between four antidepressant compounds, namely, amitriptyline hydrochloride (AMI), imipramine hydrochloride (IMI), clomipramine hydrochloride (CLO), and trimipramine maleate (TRI) acting as electron donors and two *p*-benzoquinones, namely, *p*-chloranilic acid (pCA) and 2,3-dichloro-5,6-dicyano-1,4-benzoquinone (DDQ), serving as electron acceptors. The stoichiometry of the compounds produced exhibited a consistent 1:1 ratio in all instances, as established by Job’s method. Molar absorptivities, equilibrium association constants, and several other spectroscopic properties were determined for all complexes. The developed spectrophotometric techniques were validated intra-laboratory and successfully applied for quantitative assessment of the four antidepressant active ingredients in several commercial pharmaceutical formulations. The methods are relatively simple, fast, and use readily available laboratory instrumentation, making them easily applicable by most quality control laboratories worldwide.

## 1. Introduction

Depression is a psychiatric condition that manifests through several symptoms, including but not limited to feelings of profound sadness, impaired cognitive abilities, persistent exhaustion, and contemplation of self-harm [1,2,3]. During an individual’s typical lifespan, there are three distinct phases during which this disease is most likely to be experienced. The first two phases are adolescence and early adulthood, whereas the last phase tends to emerge after the age of 60 [4]. Currently, around 300 million individuals are impacted by this phenomenon worldwide [5], which is estimated to have resulted in an annual death count of about 800,000 lives [6,7,8]. The prevalence of depression among individuals has risen as a consequence of the COVID-19 pandemic [6,9,10]. This increase can be attributed to factors such as social isolation and emotional fatigue caused by concerns about unemployment and the potential loss of loved ones [11,12].

**Figure 1:**
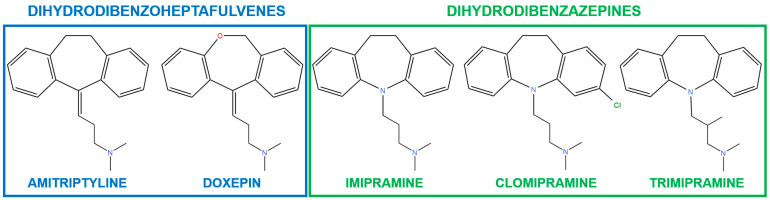
Classification of tricyclic antidepressants.

Tricyclic antidepressants (TCAs) are pharmacological agents that are commonly recommended for the treatment of major depressive disorders and anxiety disorders [13,14]. The discovery of these compounds occurred in the 1950s [15,16]. The continued utilization of these drugs in contemporary times can be attributed to their practicality and cost-efficiency [13]. These compounds are named based on their chemical structure, which comprises two benzene nuclei fused together with a heterocycle of seven atoms [15]. A pendant alkylamine chain also connects to this heterocycle [13]. The most widely recognized examples of these compounds belong to the dihydrodibenzoheptafulvene and dihydrodibenzazepine families [17] (Figure 1).

Several analytical techniques have been developed to quantify TCAs, including high-performance liquid chromatography [18,19,20,21,22], gas–liquid chromatography [2,23], cyclic voltammetry [24], capillary electrophoresis [25,26], and ultraviolet-visible (UV-VIS) spectrophotometry [27,28,29]. UV-VIS spectrophotometry is widely regarded as a favorable method for quantifying various chemical compounds due to its many benefits such as its simplicity, cost-effectiveness, little reagent usage, and adaptability [30,31,32,33]. Tricyclic antidepressants have absorption maxima in the ultraviolet (UV) region (<400 nm), owing to their multi-aromatic structures. However, it is notable that other aromatics also display maxima at such wavelengths and thus may interfere with the determination of TCAs. By moving the absorption range of tricyclic antidepressants to the visible (VIS) domain (400–760 nm), it becomes possible to enhance the accuracy of their identification and ease their quantification. One strategy to achieve this desired outcome is the employment of charge transfer (CT) molecular complexation reactions, which result in the formation of colored compounds exhibiting absorption maxima within the visible spectrum [34,35,36]. Charge transfer molecular complexes refer to molecular associations that arise from the interaction between two distinct species, namely, an electron donor and an electron acceptor [37,38,39]. Charge migration facilitates the establishment of a linkage between the two molecules, resulting in modifications of the spectral bands of either the donor or the acceptor [39,40,41]. The significance of charge transfer complexes lies in their wide-ranging utility across multiple domains, including solar energy storage, antimicrobial investigations [36,42], photocatalysis, and microemulsion systems [43,44,45].

**Figure 2:**
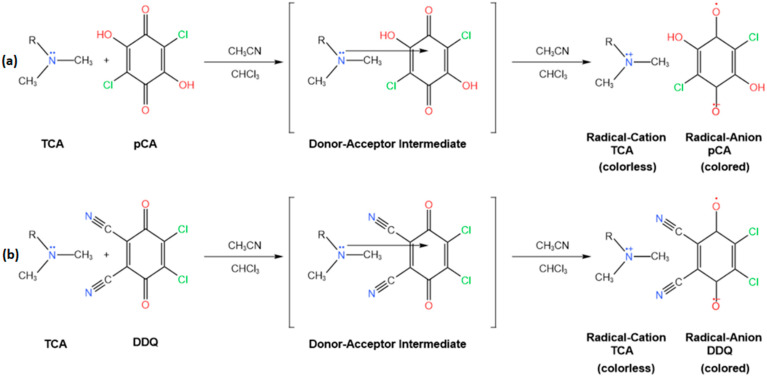
Association reactions of TCA base forms with pCA (**a**) and DDQ (**b**) in CHCl_3_:CH_3_CN 1:1.

In this study, we present the development of two spectrophotometric techniques that rely on the visible absorptions of donor–acceptor molecular complexes formed between tricyclic antidepressants and two acceptors, *p*-chloranilic acid (pCA) and 2,3-dichloro-5,6-dicyano-1,4-benzoquinone (DDQ), respectively (Figure 2). Our research includes determinations of various parameters such as the stoichiometry of the association reaction, molar absorptivities, stability (association) constants of the molecular complexes, and the analytical figures of merit of the developed method. The present study also describes the effective use of this method for assessing the concentration of the active ingredient in various pharmaceutical formulations and preparations available on the European market, as well as comparisons with other similar spectrophotometric methods.

## 2. Results and Discussion

### 2.1. Absorption Spectra of Reagents and of Resulting TCA-pCA/DDQ Complexes

**Figure 3:**
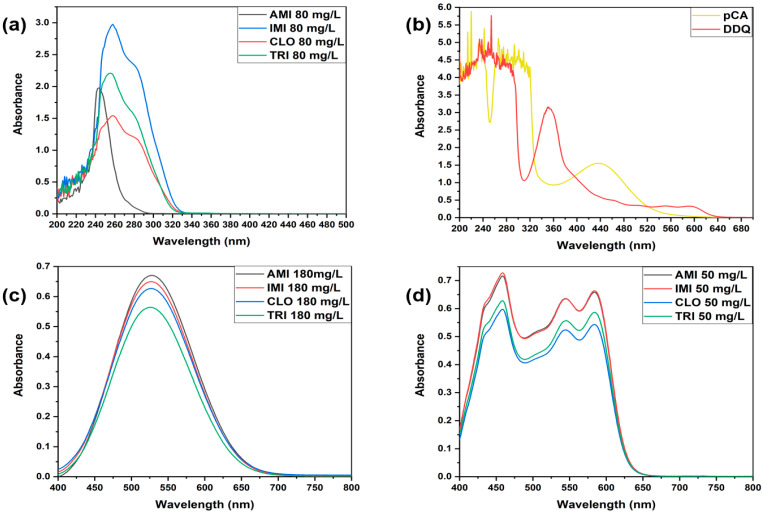
UV-VIS absorption spectra of the four TCA base forms in chloroform (**a**), pCA and DDQ in acetonitrile (**b**), TCA-pCA (**c**), and TCA-DDQ (**d**) complexes in chloroform-acetonitrile.

Antidepressant solutions in chloroform are colorless and show absorption maxima in the UV range (Figure 3a). The spectra of the *p*-benzoquinones dissolved in acetonitrile exhibit their most red-shifted local absorption maxima at 435 nm in the case of pCA and at 345 nm in the case of DDQ, respectively (Figure 3b). The interaction between the four tricyclic antidepressants in their base form (acting as electron donors) and the two *p*-benzoquinones, pCA and DDQ (acting as π electron acceptors), in a 1:1 (*v*/*v*) mixture of chloroform-acetonitrile resulted in the formation of molecular complexes that exhibited absorptions red-shifted well into the visible range, with maxima at 530 nm for all TCA-pCA and 585 nm for all TCA-DDQ complexes, respectively (Figure 3c,d).

### 2.2. The Molecular Compositions of TCA-pCA/DDQ Complexes

**Figure 4:**
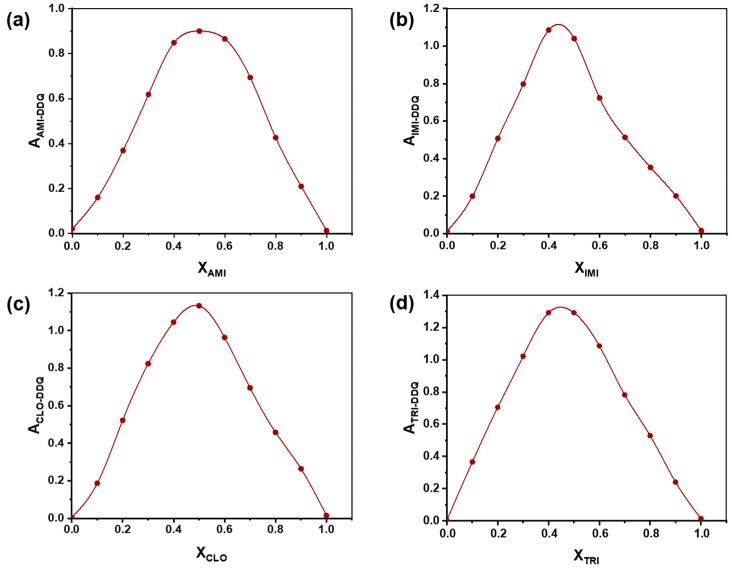
Job plots obtained for the charge transfer complexes of pCA acceptor with the base forms of amitriptyline (**a**), imipramine (**b**), clomipramine (**c**), and trimipramine (**d**) as donors.

**Figure 5:**
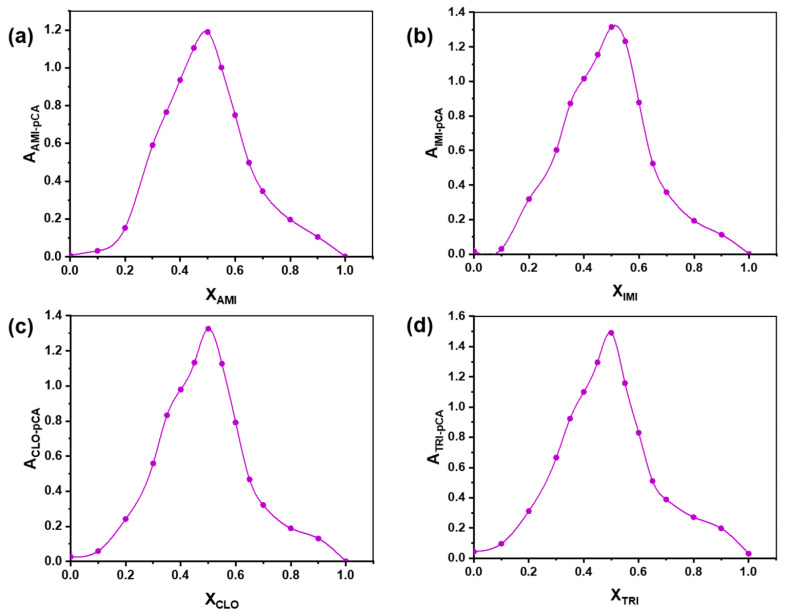
Job plots obtained for the charge transfer complexes of DDQ acceptor with the base forms of amitriptyline (**a**), imipramine (**b**), clomipramine (**c**), and trimipramine (**d**) as donors.

After having identified the characteristic absorption maxima of all molecular charge transfer complexes, Job’s method [46] was used to establish the stoichiometry of these complexes formed by TCA donors and pCA/DDQ acceptors. When continuously varying the relative ratio between donor and acceptor, while maintaining the sum of their molar concentrations constant, there is a point where the absorbance of the mixture reaches a maximum because the ratio corresponds to the exact association stoichiometry between the donor and acceptor, thus maximizing the concentration of the association complex in the mixture at equilibrium. The maximum absorbance was observed at a mole fraction of 0.5 in all cases (Figure 4a–d and Figure 5a–d), suggesting that all examined complexes exhibit a 1:1 molar ratio between their donor and acceptor constituents. Consequently, it appears that the specific natures of the donor and acceptor, among the four TCAs and two *p*-benzoquinones studied here, did not influence the stoichiometry of their association complexes.

### 2.3. Molar Absorptivities of TCA-pCA/DDQ Complexes

**Figure 6:**
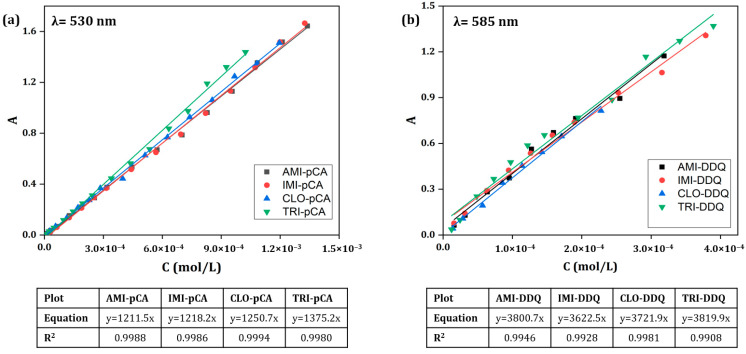
Lambert–Beer plots obtained for the charge transfer complexes of pCA (**a**) and DDQ (**b**) with the base forms of the four TCAs.

Molar absorptivities (molar extinction coefficients) were determined as the slopes of absorbance–molar concentration linear dependencies, according to Lambert–Beer law, considering the 1 cm optical path of cuvettes in all cases (Figure 6). From these data, it can be concluded that the molar absorptivities are influenced to a greater extent by the nature of the acceptor and very little by the donor, as all TCA-pCA complexes had very similar molar absorptivities in the range 1200–1400 L mol^−1^ cm^−1^, whereas those of TCA-DDQ complexes were considerably higher, ranging from ~3600 to ~3850 L mol^−1^ cm^−1^.

### 2.4. Formation (Stability) Constants of TCA-pCA/DDQ Complexes

The stability constants corresponding to the formation reactions of the complexes (*K*) were calculated according to the defining equation for an equilibrium constant:

(1) $$K=\frac{C_{C}}{\left(C_{A}-C_{C}\right)\cdot \left(C_{D}-C_{C}\right)}$$

where

- *C_C_* is the concentration of complex formed at equilibrium (mol/L), determined by dividing the measured absorbances of equimolar mixtures of donor and acceptor by the molar absorptivity of the complex and by the optical path length of the cuvette (1 cm);
- *C_D_* is the initial concentration of the TCA donor (mol/L);
- *C_A_* is the initial concentration of the pCA or DDQ acceptor, respectively (mol/L).

The high values of the formation constants obtained (Table 1), of the order 10^3^ to 10^5^, provide evidence that all ion-pair complexes possess high stability. The magnitude of this parameter is strongly dependent upon the characteristics of the acceptor employed, DDQ determining 4- to 20-fold higher *K* values than pCA when coupled to the same donor compound, which is likely due to factors such as the nature and number of electron-withdrawing substituents. In this sense, DDQ bearing the more electron-withdrawing cyano groups is a stronger acceptor than pCA, leading to enhanced charge transfer from the donor and greater electrostatic attraction of counter-ions and thus larger stability constants of resulting complexes.

### 2.5. Spectroscopic Physical Parameters of TCA-pCA/DDQ Complexes

#### 2.5.1. Oscillator Strength and Transition Dipole Moment

The oscillator strengths (*f*) and transition dipole moments (µ) of each complex were determined according to the equations [47,48]:

(2) $$f=4.32\times 10^{-9}\left[\varepsilon_{max}{∆\nu }_{\frac{1}{2}}\right]$$

(3) $$µ=0.0952{\left\lceil \frac{\varepsilon_{max}{\Delta \nu }_{\frac{1}{2}}}{{\bar{\nu }}_{max}}\right\rceil }^{1/2}$$

where

- *ε_max_* is the molar absorptivity of the CT complex at the absorption maximum;
- ${\Delta \nu }_{\frac{1}{2}}$ is the band width at half of the maximum absorbance;
- ${\bar{\nu }}_{\max}$ is the maximum absorption wavenumber.

The values corresponding to the obtained complexes are shown in Table 1. The elevated values (0.43–0.81) of oscillator strength indicate a strong interaction and robust bond between TCA and pCA or DDQ. Moreover, the determined high dipole moment values (6.92–9.98 D) suggest very pronounced charge transfer between all donors and acceptors, but more so for DDQ complexes (9.56–9.98 D) given its stronger electron-accepting nature as compared to pCA.

#### 2.5.2. Ionization Potential

Ionization potential (*I_p_*) values were calculated using the following equation [40,47,49,50]:

(4) $$I_{P}=5.76+1.53\times 10^{-4}\nu_{CT}$$

where *ν_CT_* is the frequency corresponding to the absorption maximum.

The ionization potential may be employed to evaluate the electron-donating capacity of tricyclic antidepressants. Given that all CT complexes of the four donors with each of the two acceptors had the same maximum absorption frequency, it follows that the *I_p_* values are the same for a given acceptor with all four donors (Table 1), so this parameter suggests equal donating ability of all TCAs.

#### 2.5.3. Resonance Energy

The resonance energy (*R_N_*) of the CT complexes was estimated according to the next equation [38,51,52]:

(5) $$\varepsilon_{max}=7.7\times 10^{-4}/\left[h\nu_{CT}/{|R}_{N}|-3.5\right]$$

where

- *ε_max_* is the molar absorptivity of the CT complex at the absorption maximum;
- *h* is Planck’s constant;
- *ν_CT_* is the frequency corresponding to the absorption maximum.

The values of resonance energy (Table 1) suggest the formation of very stable adducts. Furthermore, in the case of TCA-DDQ, more stable complexes are obtained as compared to TCA-pCA, as resonance energies are higher.

### 2.6. Gibbs Free Energy Change in Complex Formation Reaction

The nature of the interaction within CT complexes was examined using the standard Gibbs free energy change (Δ*G*°) for the complexation reaction, calculated according to the equation [40,53]:

(6) $${∆G}^{{}^{\circ}}=-RTlnK$$

where

- *R* is the ideal gas constant;
- *T* is the absolute temperature;
- *K* is the formation (stability) constant of the charge transfer complex.

The very negative values of the standard Gibbs free energy change (Table 1) signify that the interaction between donors and acceptors is very strong, and adduct formation is spontaneous. Once again, somewhat more negative values were generally obtained for TCA-DDQ complexes rather than TCA-pCA complexes, indicative of a greater stability of the former in comparison to the latter.

### 2.7. Validation of the Spectrophotometric Methods

#### 2.7.1. Figures of Merit

Calibration curves for determining TCAs through their reaction with pCA and DDQ, respectively, were constructed by plotting absorbances against the concentrations of standard solutions and subsequently determining the linear regression equations over the corresponding linear range of concentrations. The least squares method was employed to obtain the values of the slope and the correlation coefficients. Together with the linear range, Sandell’s sensitivity, and the limits of detection and quantification for both methods, these are reported in Table 2.

Sandell’s sensitivity was assessed as the lowest mass of complex in µg that leads to an absorbance of 0.001 in a column of solution with a cross-section equal to 1 cm^2^ [54].

The limits of detection (*LOD*) and quantification (*LOQ*) were determined using the formulae:

(7) $$LOD=\frac{3.3\times \sigma }{S}$$

(8) $$LOQ=\frac{10\times \sigma }{S}$$

where *σ* is the standard deviation of the absorbance of the blank (*n* = 20), and *S* is the slope of the calibration curve.

Both proposed methods have great sensitivity, as highlighted by their high molar absorptivity values (Table 1), low Sandell’s sensitivity, and low *LOD*/*LOQ* values (Table 2).

#### 2.7.2. Precision and Accuracy

To evaluate the intra-day precision, the methods of analysis were replicated seven times on the same day. In order to evaluate the inter-day precision, the proposed methods were replicated five times on distinct days. Both intra- and inter-day precision were determined for three different concentrations of the TCA analytes. Precision results are represented by relative standard deviations (RSD), which were consistently below 2% both intra- and inter-day (Table 3).

The evaluation of accuracy involved determining the degree of agreement between the concentrations measured by spectrophotometry and the known initial concentrations of each antidepressant used. These are reported as percentage recoveries in Table 3 and were above 98% in all cases (Table 3).

### 2.8. Comparison with Other Spectrophotometric Methods Published in the Literature

Table 4 is a survey of other spectrophotometric methods published in the literature designed for the quantitative analysis of tricyclic antidepressants, along with their figures of merit and some remarks on their limitations. Our methods are simple, fast, convenient, sensitive, reproducible, and pose a number of advantages over many of the other available options.

One major advantage in comparison to some of the other methods [55,56,57,58,59] is that the absorbance maxima of colored products lie in the visible range, as opposed to the UV. This eliminates the possible interference from other contaminants (e.g., aromatics) that may be co-extracted from the analyzed matrix and demands simpler analytical instrumentation, as spectrophotometers capable of measuring in the UV domain are much more expensive and not available worldwide to all quality control laboratories plus consumables (e.g., replacement deuterium or xenon lamps) bring an additional financial cost. Furthermore, absorbance measurements in the VIS domain may be performed using simple, cheap glass cuvettes as opposed to the much pricier quartz cuvettes.

A second advantage of our methods is the instant formation of highly absorbing colored products in ambient conditions. Many of the other published procedures require heating for several minutes at high temperatures with or without subsequent cooling to generate a stable color [60,61,62,63,64,65] or cooling to low temperatures due to limited stability of some of the reagents used [63,66].

Moreover, for the methods described in our work, color is generated by the direct reaction of the TCA donor with the *p*-benzoquinone acceptor, whereas other published methods quantify TCAs in an indirect manner, relying on deliberately added dyes that are partially bleached by the excess of a reagent not consumed in a direct reaction with the antidepressant [67,68].

Another advantage is the broad linear ranges of the two methods herein, somewhat broader in the case of the pCA option as compared to DDQ, which surpass the reported linear ranges of most other methods, as shown in Table 4.

One limitation of our described methods is linked to the fact that they require an additional pre-extraction step into chloroform, but several other methods also require extraction solvents [55,59,69]. Nevertheless, the extraction step can also be regarded as advantageous, as it constitutes a pre-purification step, eliminating insoluble possible interferences from the matrix.

**Table 4:** Other published spectrophotometric methods of analysis for tricyclic antidepressant drugs.

Reagent(s) Used	TCA	λ_max_, nm	*ε*, L mol^−1^ cm^−1^	Linear Range, µg mL^−1^	Sandell’s Sensitivity, µg cm^−2^	Remarks	Ref.
K_2_Cr_2_O_7_ + H_2_SO_4_	IMI CLO TRI	670	3.30 × 10^4^ 1.17 × 10^4^ 2.01 × 10^4^	2–14 2–25 2–25	NR *	Requires heating at 50 °C for 25 min to achieve a stable color, narrow linear range	[60]
Ammonium molybdate + H_2_SO_4_	AMI	660	2.41 × 10^3^	1–140	NR	Requires heating at 100 °C for at least 20 min to achieve stable color	[61]
Diazotized *p*-phenylenediamine dihydrochloride + H_2_SO_4_	IMI CLO TRI	565	5.86 × 10^4^ 6.89 × 10^4^ 8.01 × 10^4^	0.1–4.0 0.1–3.6 0.1–3.2	0.005 0.005 0.005	Requires cooling to temperatures below 5 °C for preparation of the diazotized amine, narrow linear range	[66]
Niobium (V) thiocyanate + HCl+ butanol extractant	AMI	360	2.17 × 10^4^	1–12	NR	Absorbance maximum is in the UVA domain, narrow linear range, extractive method	[55]
Molybdenum (V) thiocyanate + HCl+ CH_2_Cl_2_ extractant	AMI	470	1.09 × 10^4^	2–30	0.1181	Narrow linear range, extractive method	[69]
Excess Br_2_ + Methyl red	AMI IMI CLO	520	0.65 × 10^5^ 1.41 × 10^5^ 1.93 × 10^5^	0.0–2.5 0.0–1.4 0.0–1.4	0.0048 0.0022 0.0018	Indirect method based on partial bleaching of methyl red color by excess Br_2_ after TCA bromination, narrow linear range	[67]
Excess Br_2_ + Eriochrome blue black R	IMI CLO	530	1.57 × 10^4^ 1.62 × 10^4^	0.0–9.0 0.0–10.0	0.0202 0.0216	Indirect method based on partial bleaching of erio R color by excess Br_2_ after TCA bromination, narrow linear range	[68]
Fe(III) + NH_4_SCN + HNO_3_	AMI	460	2.82 × 10^3^	1.0–10.0	NR	Narrow linear range	[29]
Ce(SO_4_)_2_ + HClO_4_	TRI	620	3.0 × 10^4^	0.4–10.0	NR	Narrow linear range	[62]
KIO_4_ + H_2_SO_4_	TRI	670	1.1 × 10^4^	4–42	NR	Requires heating at 75 °C for 30 min to achieve stable color	[62]
β-Cyclodextrine + PEG	AMI	242	2.2 × 10^4^	0.1–1.0	NR	Absorbance maximum is in the UVC domain, narrow linear range	[56]
I_2_	IMI	366	2.05 × 10^3^	2.0–25	0.0141	Absorbance maximum is in the UVA domain	[57]
3-methylbenzothiazolin-2-one hydrazone + ammonium iron (III) sulfate + HCl	IMI TRI CLO	630 630 620	8.15 × 10^4^ 7.80 × 10^4^ 4.23 × 10^4^	0.5–4 1–5 1–8	NR	Requires heating at 30 °C for 15 min, narrow linear range	[63]
I_2_	TRI	292	7.1 × 10^4^	1–5	0.0057	Absorbance maximum is in the UVC domain, narrow linear range, color obtained after 30 min and stable for only 30 min	[58]
Chloranil	TRI	220	1.6 × 10^4^	5–50	0.0256	Absorbance maximum is in UVC domain, stable color is obtained after 30 min	[58]
2,2′-bipyridine + CH_3_COOH	IMI CLO TRI	530	6.59 × 10^4^ 6.55 × 10^4^ 6.80 × 10^4^	0.2–2.4 0.2–3.2 0.2–2.0	0.0048 0.0022 0.0018	Requires boiling the solution for 30 min to achieve stable color, narrow linear range, applicable only to dibenzazepines	[64]
Diazotized *p*-nitroaniline + HCl	IMI TRI CLO	575	3.3 × 10^4^ 4.8 × 10^4^ 1.9 × 10^4^	1–10 1–10 3–20	NR	Requires storing the solution in ice bath and must be freshly prepared every 5 h, requires heating at 75 °C for 20 min and cooling to achieve color, narrow linear range	[63]
Bromocresol green + CH_2_Cl_2_ extractant	CLO	402	1.11 × 10^4^	1.65–34.78	0.03	Absorbance maximum almost in the UV, extractive method	[59]
Ammonium molybdate + CH3COOH	CLO	712	7.11 × 10^3^	1–250	NR	Requires heating at 90 °C for 35 min and cooling to achieve stable color	[65]
pCA/CH_3_CN + CHCl_3_	AMI IMI CLO TRI	530	1.21 × 10^3^ 1.22 × 10^3^ 1.25 × 10^3^ 1.38 × 10^3^	5–420 5–420 5–420 5–420	0.2591 0.2601 0.2809 0.2985	Extractive method	This work
DDQ/CH_3_CN + CHCl_3_	AMI IMI CLO TRI	585	3.80 × 10^3^ 3.62 × 10^3^ 3.72 × 10^3^ 3.95 × 10^3^	5-100 5–120 5–80 5–140	0.0826 0.0875 0.0944 0.1039	Extractive method	This work

* NR: not reported.

### 2.9. Application of the Proposed Spectrophotometric Methods

The two spectrophotometric approaches were employed to determine the tricyclic antidepressants under investigation from pharmaceutical dosage forms. The procedure was conducted in triplicate. Following the extraction process using chloroform and subsequent conversion to the base form, the general determination procedure was implemented. The results are presented in Table 5 and are in very good agreement with the amounts reported by the manufacturers. Both methods performed equally well, retrieving 98 to 100% of the expected amounts from all pharmaceutical products tested.

## 3. Materials and Methods

### 3.1. Chemicals

Amitriptyline hydrochloride (AMI, ≥98%), clomipramine hydrochloride (CLO, ≥98%), imipramine hydrochloride (IMI, ≥99%), trimipramine maleate salt (TRI, ≥98%), *p*-chloranilic acid (pCA, ≥98%), 2,3-dichloro-5,6-dicyano-*p*-benzoquinone (DDQ, ≥98%), sodium carbonate (≥99%), and sodium sulfate (≥99%, anhydrous) were acquired from Merck (Darmstadt, Germany). Acetonitrile (≥99.9%, HPLC grade) and chloroform (≥99.8%, GC grade) were acquired from Scharlab S.L. (Barcelona, Spain). Doubly distilled water from an in-house distillation setup was used for the preparation of aqueous solutions.

### 3.2. Pharmaceutical Products

The various pharmaceutical formulations comprising the aforementioned antidepressants as active ingredients were purchased from local drug stores in Romania and France. They are denoted as “Commercial form” 1–4 throughout the paper. The dosage forms consisted of film-coated tablets in all cases, with different active substances and different declared concentrations, as shown in Table 6, which also includes listings of all other excipients contained according to the commercial labels of the products.

### 3.3. Analytical Instrumentation

An analytical balance Kern ABD 200-4 (KERN Gmbh, Düren, Germany) was used for weighting samples with a precision of ±0.0001 g. An Evolution 220 (Thermo Scientific Inc., Waltham, MA, USA) double-beam UV-VIS spectrophotometer, with a spectral range 190–1100 nm and equipped with quartz cuvettes with a 10 mm path length, was used for all absorbance measurements.

### 3.4. Preparation of Stock Solutions

Stock solutions (2 g/L) of antidepressants in their as-received (i.e., protonated/salt) forms were made by accurately weighing the corresponding amounts of TCAs on the analytical balance, followed by dissolution into chloroform as solvent. DDQ (0.05%, by weight) and pCA (0.20%, by weight) solutions were prepared similarly using acetonitrile as solvent. A sodium carbonate aqueous solution (0.6 M) was prepared using doubly distilled water.

### 3.5. Preparation of Antidepressant Base form Standard Solutions

In a typical procedure, a volume of 25 mL from the stock solution of the antidepressant salt form was carefully poured into a separating funnel. Subsequently, 50 mL of the aqueous sodium carbonate solution (0.6 M) was added to the separating funnel. The funnel was vigorously shaken for a duration of three minutes. The two immiscible phases were allowed to separate, and then the chloroform layer was collected and dried over anhydrous sodium sulfate, thus yielding the standard solution of the TCA base form. Further dilutions provided standard solutions with various concentrations of TCA base.

### 3.6. Extraction of Pharmaceutically Active Ingredients from Commercial Tablets and Preparation of Their Base form Solutions

Commercial film-coated tablets were first weighed, then the film was removed completely by scratching it off with metal blades. The number of tablets used in a typical extraction experiment differed based on their expected active compound content: four tablets in the case of AMI and CLO, ten tablets for IMI, and two tablets in the case of TRI. Subsequently, the uncoated tablets were ground to a fine powder using an agate mortar and a pestle. The resulting powder was weighed and subjected to extraction of the active compound using 25 mL of chloroform. Insoluble excipients from the ground tablet were filtered off and washed on the filter with an additional 25 mL of chloroform, which were pooled together with the first 25 mL of filtrate. To obtain the base forms of commercial TCAs, half (25 mL) of the chloroform extract was added to a separation funnel and the procedure in Section 3.4 repeated, yielding the base form solutions in chloroform of TCAs retrieved from commercial pharmaceutical formulations. Such a protocol would be expected to yield chloroform extracts with TCA concentrations around 1.6–2.0 g/L, similar to the stock solutions of antidepressant standards mentioned above.

### 3.7. General Procedure for Spectrophotometric Measurements

A precise quantity of antidepressant solution in its base form was introduced into a 5 mL volumetric flask. Subsequently, 1 mL of pCA or DDQ and 1.5 mL of acetonitrile were added into the solution, followed by filling the remaining volume with CHCl_3_ up to the mark to ensure, for consistency purposes, equal volumes of chloroform and acetonitrile in all experiments. Then, the flask was well homogenized. The UV-VIS spectrophotometer was used to measure the absorbances of the resulting colored solution at the characteristic wavelengths for pCA and DDQ complexes, i.e., 530 nm and 585 nm, respectively, against blanks prepared using only the same amounts of either pCA or DDQ, equal volumes of acetonitrile and chloroform, but no TCA.

### 3.8. Molar Ratio Determination for the Charge Transfer Complexes TCA-pCA and TCA-DDQ

The stoichiometric combination ratio of molecular charge transfer complexes between TCAs and pCA and, respectively, TCAs and DDQ was determined by applying Job’s method, also known as the continuous variation method [46]. This method consists of the preparation of a series of solutions in which the mole fraction of the TCA in base form was gradually increased from 0 to 1, in 0.1 increments, whereas the mole fraction of pCa or DDQ decreased simultaneously at the same pace, from 1 to 0. The absorbances were measured at the wavelengths corresponding to the absorption maxima, specifically 530 nm for TCA-pCA and 585 nm for TCA-DDQ.

## 4. Conclusions

The charge transfer reaction between several compounds belonging to the tricyclic antidepressant class and pCA or DDQ was investigated with the aim of developing two new VIS spectrophotometric methods that are simple, fast, precise, and accurate. The experimental results indicated that all of the complexes studied exhibited a stoichiometry of 1:1 between TCA and pCA or DDQ, respectively. The molecular complexes were produced spontaneously and provide remarkable stability, as evidenced by their high values of equilibrium formation constants and oscillator strengths, as well as the negative values of Gibbs free energy of formation. The developed analytical methods were characterized in terms of their characteristic figures of merit and were successfully applied for the determination of TCA active ingredients found in some commercial drug formulations typically tested by quality control laboratories in the pharmaceutical industry. The same methods hold promise for applicability in the analysis of other types of real-world samples that may contain TCAs, for example, wastewaters or aqueous biological fluids.

## Figures and Tables

**Table 1:** Thermodynamic and spectroscopic physical parameters of TCA-pCA and TCA-DDQ complexes.

TCA	*ε*, L mol^−1^ cm^−1^	*K*, L mol^−1^	*f*	µ, Debyes	*I_p_*, eV	*R_N_*, eV	Δ*G*°, kJ mol^−1^
AMI	1211.5	10,108.47	0.43	6.92	8.64	0.035	−22.86
IMI	1218.2	10,669.97	0.43	6.92		0.035	−22.99
CLO	1250.7	9209.99	0.44	7.01		0.036	−28.63
TRI	1375.2	3108.26	0.49	7.35		0.039	−19.93
AMI	3800.7	41,929.36	0.78	9.79	8.37	0.090	−26.38
IMI	3622.5	211,733.40	0.74	9.56		0.086	−30.40
CLO	3721.9	84,317.50	0.76	9.69		0.089	−28.12
TRI	3952.4	65,812.46	0.81	9.98		0.093	−27.50

**Table 2:** Figures of merit for the two spectrophotometric methods developed.

Parameters	AMI	IMI	CLO	TRI
TCA-pCA				
λ_max_, nm	530	530	530	530
Linear range, µg∙mL^−1^	5–420	5–420	5–420	5–420
R^2^	0.9971	0.9966	0.9985	0.9953
Slope S, mL∙µg^−1^	0.0039	0.0038	0.0036	0.0033
Sandell’s sensitivity, µg∙cm^−2^	0.2591	0.2601	0.2809	0.2985
*LOD*, µg∙mL^−1^	0.83	0.85	0.90	0.98
*LOQ*, µg∙mL^−1^	2.52	2.58	2.73	2.97
TCA-DDQ				
λ_max_, nm	585	585	585	585
Linear range, µg∙mL^−1^	5–100	5–120	5–80	5–140
R^2^	0.9946	0.9928	0.9981	0.9915
Slope S, mL∙µg^−1^	0.0121	0.0114	0.0106	0.0096
Sandell’s sensitivity, µg∙cm^−2^	0.0826	0.0875	0.0944	0.1039
*LOD*, µg∙mL^−1^	0.39	0.41	0.44	0.19
*LOQ*, µg∙mL^−1^	1.18	1.28	1.34	1.48

**Table 3:** Recoveries and intra- and inter-day precision data for the two spectrophotometric methods at three TCA concentration levels.

TCA	Concentration, mg/L	Intra-Day (*n* = 7) Recovery ± RSD, %	Inter-Day (*n* = 5) Recovery ± RSD, %
TCA-pCA			
AMI	5	99.85 ± 0.70	99.78 ± 0.82
	210	99.93 ± 0.25	99.89 ± 0.35
	420	99.96 ± 0.18	99.91 ± 0.43
IMI	5	99.94 ±0.85	99.97 ± 0.41
	210	99.98 ± 0.15	99.87 ±0.35
	420	99.97 ± 0.52	99.82 ±0.73
CLO	5	99.13 ± 0.81	99.18 ± 0.61
	210	99.27 ± 0.64	99.01 ± 0.95
	420	99.63 ± 0.93	99.72 ± 0.87
TRI	5	99.90 ± 0.33	99.32 ± 0.39
	210	99.83 ± 0.67	99.02 ± 0.81
	420	99.87 ± 0.78	98.99 ± 0.98
TCA-DDQ			
AMI	5	100.02 ± 0.38	99.18 ± 0.95
	50	99.75 ± 0.57	99.23 ± 1.02
	100	99.88 ± 0.39	99.61 ± 0.99
IMI	5	99.81 ± 0.43	99.56 ± 1.13
	60	99.16 ± 0.87	99.40 ± 1.65
	120	99.03 ± 0.99	99.71 ± 0.72
CLO	5	98.39 ± 0.72	99.01 ± 1.34
	40	99.09 ± 0.93	99.23 ± 1.72
	80	99.13 ± 0.55	99.41 ± 1.06
TRI	5	98.77 ± 1.12	98.32 ± 1.21
	80	99.19 ± 1.28	99.05 ± 1.04
	160	98.36 ± 0.93	99.12 ± 0.97

**Table 5:** Results obtained for TCA quantifications from pharmaceutical dosage forms using the two spectrophotometric methods.

Commercial Form	Active Substance	Amount Expected, mg/Dosage Form	Amount Found ± SD, mg/Dosage Form	Recovery ± RSD, %
TCA-pCA				
Commercial form 1	AMI	25.00	24.95 ± 0.08	99.80 ± 0.32
Commercial form 2	IMI	10.00	9.91 ± 0.02	99.12 ± 0.19
Commercial form 3	CLO	25.00	24.94 ± 0.16	99.77 ± 0.64
Commercial form 4	TRI	40.00	39.17 ± 0.08	97.93 ± 0.21
TCA-DDQ				
Commercial form 1	AMI	25.00	24.98 ± 0.35	99.92 ± 1.40
Commercial form 2	IMI	10.00	9.97 ± 0.05	99.70 ± 0.50
Commercial form 3	CLO	25.00	24.89 ± 0.27	99.56 ± 1.08
Commercial form 4	TRI	40.00	40.03 ± 0.04	100.07 ± 0.16

**Table 6:** Active ingredient content and excipients of commercial pharmaceutical dosage forms analyzed.

Commercial Form	Active Substance	Amount of Active Substance Declared, mg/Dosage Form	Excipients
Commercial form 1	AMI	25.00	Film: hypromellose, hydroxypropyl cellulose, talcum, lactose, stearic acid, titanium dioxide, carmoisine aluminum lake 20–26%, quinoline yellow aluminum lake 20–24% Core: lactose, polyvinylpyrrolidone K30, starch, talcum, magnesium stearate
Commercial form 2	IMI	10.00	Film: hypromellose, pyrrolidone-vinyl acetate copolymer, microcrystalline cellulose, macrogol 8000, polyvinylpyrrolidone K30, talcum, sucrose, titanium dioxide, red iron oxide dispersed in titanium dioxide 30/70 Core: anhydrous colloidal silica, anhydrous glycerol, lactose, magnesium stearate, starch, talcum, stearic acid
Commercial form 3	CLO	25.00	Film: hypromellose 2910, pyrrolidone-vinyl acetate copolymer, talcum, sucrose, macrogol 8000, polyvinylpyrrolidone, yellow iron oxide, titanium dioxide Core: lactose, glycerol 85%, magnesium stearate, starch, silica, talcum, stearic acid
Commercial form 4	TRI	40.00	Film: hypromellose, pyrrolidone-vinyl acetate copolymer, talcum, sucrose, macrogol 400, polyvinylpyrrolidone, yellow iron oxide, titanium dioxide Core: lactose, glycerol, calcium hydrogen phosphate, magnesium stearate, starch, silica, talcum, stearic acid

## Data Availability

All data are contained within the article.

## References

[1] Mehdi S., Manohar K., Shariff A., Wani S.U.D., Almuqbil M., Alshehri S., Shakeel F., Imam M.T., Krishna K.L. (2022). Analysis of Antidepressants Utilization for Patients Visiting Psychiatric Out-Patient Clinic in a Tertiary Care Hospital. Healthcare.

[2] Soares S., Rosado T., Barroso M., Gallardo E. (2021). New Method for the Monitoring of Antidepressants in Oral Fluid Using Dried Spot Sampling. Pharmaceuticals.

[3] Matic T., Pregelj P., Sadikov A., Rus Prelog P. (2022). Depression, Anxiety, Stress, and Suicidality Levels in Young Adults Increased Two Years into the COVID-19 Pandemic. Int. J. Environ. Res. Public Health.

[4] Arias-de la Torre J., Ronaldson A., Prina M., Matcham F., Pinto Pereira S.M., Hatch S.L., Armstrong D., Pickles A., Hotopf M., Dregan A. (2021). Depressive symptoms during early adulthood and the development of physical multimorbidity in the UK: An observational cohort study. Lancet Healthy Longev..

[5] Hudson D., Collins-Anderson A. (2022). Understanding Perceptions of Depression and Depression Care across Culture and Context. Int. J. Environ. Res. Public Health.

[6] Ciuca M.D., Racovita R.C. (2023). Curcumin: Overview of Extraction Methods, Health Benefits, and Encapsulation and Delivery Using Microemulsions and Nanoemulsions. Int. J. Mol. Sci..

[7] Moitra M., Santomauro D., Degenhardt L., Collins P.Y., Whiteford H., Vos T., Ferrari A. (2021). Estimating the risk of suicide associated with mental disorders: A systematic review and meta-regression analysis. J. Psychiatr. Res..

[8] Polak M., Nowicki G.J., Naylor K., Piekarski R., Slusarska B. (2022). The Prevalence of Depression Symptoms and Their Socioeconomic and Health Predictors in a Local Community with a High Deprivation: A Cross-Sectional Studies. Int. J. Environ. Res. Public Health.

[9] Zalewska A., Galczyk M., Van Damme-Ostapowicz K. (2022). Level of Depression during the COVID-19 Pandemic in Poland—A Cross-Sectional Study. Healthcare.

[10] Aveiro-Robalo T.R., Garlisi-Torales L.D., Chuman-Sanchez M., Pereira-Victorio C.J., Huaman-Garcia M., Failoc-Rojas V.E., Valladares-Garrido M.J. (2022). Prevalence and Associated Factors of Depression, Anxiety, and Stress in University Students in Paraguay during the COVID-19 Pandemic. Int. J. Environ. Res. Public Health.

[11] Balakrishnan V., Ng K.S., Kaur W., Govaichelvan K., Lee Z.L. (2022). COVID-19 depression and its risk factors in Asia Pacific—A systematic review and meta-analysis. J. Affect. Disord..

[12] Stephenson E., O’Neill B., Kalia S., Ji C., Crampton N., Butt D.A., Tu K. (2022). Effects of COVID-19 pandemic on anxiety and depression in primary care: A retrospective cohort study. J. Affect. Disord..

[13] Manousi N., Samanidou V.F. (2019). Applications of Gas Chromatography for the Analysis of Tricyclic Antidepressants in Biological Matrices. Separations.

[14] Achenbach J., Saft C., Faissner S. (2021). Longitudinal Evaluation of the Effect of Tricyclic Antidepressants and Neuroleptics on the Course of Huntington’s Disease-Data from a Real World Cohort. Brain Sci..

[15] Asensi-Canto A., Lopez-Abellan M.D., Castillo-Guardiola V., Hurtado A.M., Martinez-Penella M., Luengo-Gil G., Conesa-Zamora P. (2022). Antitumoral Effects of Tricyclic Antidepressants: Beyond Neuropathic Pain Treatment. Cancers.

[16] Wu Q., Xu Y., Bao Y., Alvarez J., Gonzales M.L. (2020). Tricyclic Antidepressant Use and Risk of Fractures: A Meta-Analysis of Cohort Studies through the Use of Both Frequentist and Bayesian Approaches. J. Clin. Med..

[17] Enescu L. (2005). Medicamente, Sinteze si Utilizari.

[18] Malfara W.R., Bertucci C., Costa Queiroz M.E., Dreossi Carvalho S.A., Pires Bianchi Mde L., Cesarino E.J., Crippa J.A., Costa Queiroz R.H. (2007). Reliable HPLC method for therapeutic drug monitoring of frequently prescribed tricyclic and nontricyclic antidepressants. J. Pharm. Biomed. Anal..

[19] Dziurkowska E., Wesolowski M. (2023). Isolation of Antidepressants and Their Metabolites from Saliva Using Supported Liquid Extraction (SLE). Biomedicines.

[20] Breaud A.R., Harlan R., Kozak M., Clarke W. (2009). A rapid and reliable method for the quantitation of tricyclic antidepressants in serum using HPLC-MS/MS. Clin. Biochem..

[21] Poklis J.L., Wolf C.E., Goldstein A., Wolfe M.L., Poklis A. (2012). Detection and quantification of tricyclic antidepressants and other psychoactive drugs in urine by HPLC/MS/MS for pain management compliance testing. J. Clin. Lab. Anal..

[22] Fisichella M., Morini L., Sempio C., Groppi A. (2014). Validation of a multi-analyte LC-MS/MS method for screening and quantification of 87 psychoactive drugs and their metabolites in hair. Anal. Bioanal. Chem..

[23] Racovita R.C., Ciuca M.D., Catana D., Comanescu C., Ciocirlan O. (2023). Microemulsions of Nonionic Surfactant with Water and Various Homologous Esters: Preparation, Phase Transitions, Physical Property Measurements, and Application for Extraction of Tricyclic Antidepressant Drugs from Aqueous Media. Nanomaterials.

[24] Guzinski M., Lindner E., Pendley B., Chaum E. (2022). Electrochemical sensor for tricyclic antidepressants with low nanomolar detection limit: Quantitative Determination of Amitriptyline and Nortriptyline in blood. Talanta.

[25] Madej K., Wozniakiewicz M., Karabinowska K. (2012). Capillary electrophoresis screening method for six tricyclic antidepressants in human serum. Acta Pol. Pharm. Drug Res..

[26] Dell’Aquila C. (2002). Separation of tricyclic antidepressants by capillary zone electrophoresis with N,N,N′,N′-tetramethyl-1,3-butanediamine (TMBD) as an effective electrolyte additive. J. Pharm. Biomed. Anal..

[27] Rahman N., Sameen S., Kashif M. (2016). Spectroscopic study of charge transfer complexation between doxepin and π–acceptors and its application in quantitative analysis. J. Mol. Liq..

[28] Azmi S.N.H., Al-Masrouri Z.N., Al-Lamki I.R., Al-Jabri A.K., Rahman N., Nasir M., Abdelrahman K., Fnais M.S., Alam M. (2022). Development and validation of spectrophotometric method for determination of imipramine hydrochloride in tablets (solid materials). J. King Saud Univ. Sci..

[29] Soni P., Sinha D., Patel R. (2013). Simple, Rapid and Sensitive UV-Visible Spectrophotometric Method for Determination of Antidepressant Amitriptyline in Pharmaceutical Dosage Forms. J. Spectrosc..

[30] Kirova G.K., Velkova Z.Y., Delchev V.B., Gavazov K.B. (2023). Vanadium-Containing Anionic Chelate for Spectrophotometric Determination of Hydroxyzine Hydrochloride in Pharmaceuticals. Molecules.

[31] Gavazov K.B., Racheva P.V., Milcheva N.P., Divarova V.V., Kiradzhiyska D.D., Genc F., Saravanska A.D. (2022). Use of a Hydrophobic Azo Dye for the Centrifuge-Less Cloud Point Extraction-Spectrophotometric Determination of Cobalt. Molecules.

[32] Divarova V.V., Saravanska A., Toncheva G., Milcheva N., Delchev V.B., Gavazov K. (2022). Spectrophotometric Determination of Molybdenum(VI) as a Ternary Complex with 4-Nitrocatechol and Benzalkonium Chloride. Molecules.

[33] Gavazov K.B., Racheva P.V., Saravanska A.D., Toncheva G.K., Delchev V.B. (2023). Extractive Spectrophotometric Determination and Theoretical Investigations of Two New Vanadium(V) Complexes. Molecules.

[34] Shehab O.R., Mansour A.M. (2013). Charge transfer complexes of 2-arylaminomethyl-1H-benzimidazole with 2,3-dichloro-5,6-dicyano-1,4-benzoquinone: Experimental and DFT studies. J. Mol. Struct..

[35] Rodrigues de Carvalho F., da Silva F., de Lima R., Correia Bellotto A., de Souza V.R., Caetano W., Politi M.J., Hioka N., Coutinho K. (2022). Spectrophotometric studies of charge-transfer complexes formed with ions N,N’-alkyldiyl-bis(pyridinium) derivatives and iodide. Spectrochim. Acta A Mol. Biomol. Spectrosc..

[36] Niranjani S., Venkatachalam K. (2020). Synthesis, spectroscopic, thermal, structural investigations and biological activity studies of charge-transfer complexes of atorvastatin calcium with dihydroxy-p-benzoquinone, quinalizarin and picric acid. J. Mol. Struct..

[37] Meesala G., Syeda A.H., Varukolu M., Tigulla P. (2022). The Charge Transfer Complex between 2, 3-diamino-5-bromopyridine and Chloranilic acid: Preparation, Spectroscopic Characterization, DNA binding, and DFT/PCM analysis. J. Indian Chem. Soc..

[38] Messiad H., Hamamdia F.Z., Belguidoum K., Lemouari N., Amira-Guebailia H. (2022). Synthesis and spectroscopic characterization of charge transfer complexes of the donor hesperidin and π-acceptors; 2,3-dichloro-5,6-dicyano-1,4- benzoquinone and tetracyanoethylene. J. Mol. Struct..

[39] Al-Hazmi G.H., Hassanien A.M., Atta A.A., Refat M.S., Saad H.A., Shakya S., Adam A.M.A. (2022). Supramolecular charge-transfer complex generated by the interaction between tin(II) 2,3-naphtalocyanine as a donor with DDQ as an acceptor: Spectroscopic studies in solution state and theoretical calculations. J. Mol. Liq..

[40] AlRabiah H., Abdel-Aziz H.A., Mostafa G.A.E. (2019). Charge transfer complexes of brucine with chloranilic acid, 2,3-dichloro-5,6-dicyano-1,4-benzoquinone and tetracyanoquinodimethane: Synthesis, spectroscopic characterization and antimicrobial activity. J. Mol. Liq..

[41] Mostafa G.A.E., Yousef T.A., ElGamal A.A., Homoda A.M.A., AlRabiah H. (2022). Tamoxifen charge transfer complexes with 2,3-dichloro-5,6-dicyano-1,4-benzoquinone and 7,7,8,8-tetracyanoquinodimethan: Synthesis, spectroscopic characterization and theoretical study. Bioorg. Chem..

[42] Mihalache M., Oprea O., Vasile B.Ş., Guran C., Ardelean I.L. (2018). Synthesis, Characterization and Biological Activity of Composite Combinations of Cu (II), Fe (III) and Mn (III) with A-Ketoglutaric Acid and 1-(O-Tolyl) Biguanide. Univ. Politeh. Buchar. Sci. Bull. Ser. B Chem. Mater. Sci..

[43] Alghanmi R.M., Al-Attas A.S., Habeeb M.M. (2013). Spectrophotometric study of the charge transfer complex between 2-amino-4-picoline with chloranilic acid. J. Mol. Struct..

[44] Manojkumar P., Mahipal V., Suresh G., Venkatesh N., Ramesh M., Parthasarathy T. (2022). Exploring Interaction Dynamics of Designed Organic Charge Transfer Complex of 6-Aminoindole and Chloranilic Acid: Spectrophotometric, Characterization, Computational, Antimicrobial, and DNA Binding Properties. J. Mol. Struct..

[45] Ali M.M., Ali M., Gaballa A.S., El-Korashy S.A., Teleb S.M. (2021). Preparation, spectroscopic, characterizations and biological studies of new charge transfer complexes formed between fluconazole drug with various acceptors. Bioorg. Chem..

[46] Job P. (1928). Formation and Stability of Inorganic Complexes in Solution. Ann. Chim..

[47] Pandeeswaran M., Elango K.P. (2006). Solvent effect on the charge transfer complex of oxatomide with 2,3-dichloro-5,6-dicyanobenzoquinone. Spectrochim. Acta A Mol. Biomol. Spectrosc..

[48] Nampally V., Palnati M.K., Baindla N., Varukolu M., Gangadhari S., Tigulla P. (2022). Charge Transfer Complex between O-Phenylenediamine and 2, 3-Dichloro-5, 6-Dicyano-1, 4-Benzoquinone: Synthesis, Spectrophotometric, Characterization, Computational Analysis, and its Biological Applications. ACS Omega.

[49] Aloisi G.G., Pignataro S. (1973). Molecular complexes of substituted thiophens with σ and π acceptors. Charge transfer spectra and ionization potentials of the donors. J. Chem. Soc. Faraday Trans. 1 Phys. Chem. Condens. Phases.

[50] Sundarpal V., Kanth B.S., Rajitha N., Yadagiri B. (2023). Synthesis, spectroscopic characterization, DNA binding and DFT/PCM calculations of new Hydrogen-bonded charge transfer complex between 4-dimethylaminopyridine and Chloranilic acid. Results Chem..

[51] Briegleb G., Czekalla J. (1960). Intensity of electron transition bands in electron donator–acceptor complexes. Z. Physik. Chem. (Frankf.).

[52] Abbu V., Nampally V., Baindla N., Tigulla P. (2019). Stoichiometric, Thermodynamic and Computational DFT Analysis of Charge Transfer Complex of 1-Benzoylpiperazine with 2, 3-Dichloro-5, 6-Dicyano-1, 4-benzoquinone. J. Solut. Chem..

[53] Shehab O.R., AlRabiah H., Abdel-Aziz H.A., Mostafa G.A.E. (2018). Charge-transfer complexes of cefpodoxime proxetil with chloranilic acid and 2,3-dichloro-5,6-dicyano-1,4-benzoquinone: Experimental and theoretical studies. J. Mol. Liq..

[54] Shetty D.N., Narayana B., Samshuddin S. (2012). Sensitive methods for the spectrophotometric determinations of some antimalarial drugs. J. Chem. Pharm. Res..

[55] Misiuk W., Puzanowska-Tarasiewicz H. (2002). Spectrophotometric Determination of Some Antidepressant Drugs. Anal. Lett..

[56] Soni P., Sar S.K., Patel R. (2012). New Approach for the Determination of Tricyclic Antidepressant Amitriptyline Using beta-Cyclodextrin-PEG System via Spectrophotomerty. J. Anal. Sci. Methods Instrum..

[57] Susmitha K., Thirumalachary M., Vinod Kumar T., Venkateshwarlu G. (2013). Spectrophotometric determination of imipramine HCl in pure and pharmaceutical forms. Der Pharma Chem..

[58] Al-Badr A., Mostafa G.A.H. (2014). Spectrophotometric Determination of Trimipramine in Tablet Dosage Form via Charge Transfer Complex Formation. Trop. J. Pharm. Res..

[59] Ali E.A., Adawy A.M., El-Shahat M.F., Amin A.S. (2016). Simple spectrophotometric methods for determination of fluoxetine and clomipramine hydrochlorides in dosage forms and in some post-mortem biological fluids samples. Egypt. J. Forensic Sci..

[60] Mohamed H., Hassan H.Y., Mohamed A.-M.I., Hussein S.A. (1992). Spectrophotometric Determination of Some Dibenzazepines. Anal. Lett..

[61] Aman T., Kazi A.A., Hussain M.I., Firdous S., Khan I.U. (2000). Spectrophotometry Determination of Amitriptyline-HCl in Pure and Pharmaceutical Preparations. Anal. Lett..

[62] Starczewska B., Puzanowska-Tarasiewicz H. (1998). Spectrophotometric Determination of Trimipramine Using Cerium(IV) Sulphate(VI) and Potassium Iodate(VII). Anal. Lett..

[63] Hussein S.A., El-Kommos M.E., Hassan H.Y., Mohamed A.-M.I. (1989). Spectrophotometric determination of some dibenzazepine drugs by electrophilic coupling. Talanta.

[64] Syeda A., Mahesh H.R., Syed A.A. (2005). 2,2’-Bipyridine as a new and sensitive spectrophotometric reagent for the determination of nanoamounts of certain dibenzazepine class of tricyclic antidepressant drugs. Farmaco.

[65] Saif M.J., Anwar J., Nawaz G. (2014). New sensitive spectrophotometric method for the determination of clomipramine-HCl in pure form and pharmaceutical preparations. J. Anal. Chem..

[66] Nagaraja P., Silwadi M.F., Syed A.A. (2000). Sensitive Spectrophotometric Determination of Some Dibenzazepine Drugs with Diazotized P-Phenylenediamine Dihydrochloride. Anal. Lett..

[67] Deepakumari H.N., Prashanth M.K., Kumar B.C.V., Revanasiddappa H.D. (2015). Highly Sensitive and Validated Spectrophotometric Technique for the Assay of Some Antidepressant Drugs. J. Appl. Spectrosc..

[68] Deepakumari H.N., Prashanth M.K., Revanasiddappa H.D. (2014). Antidepressant Drugs: Highly Sensitive and Validated Spectrophotometric Technique. J. Chil. Chem. Soc..

[69] Mohamed G.G., Nour El-Dien F.A., Khalil S.M., Mohamed N.A. (2006). Spectrophotometric determination of trazodone, amineptine and amitriptyline hydrochlorides through ion-pair formation with molybdenum and thiocyanate. Spectrochim. Acta A Mol. Biomol. Spectrosc..

